# Magnetization treatment effect on some physical and biological characteristics of saline irrigation water

**DOI:** 10.1038/s41598-025-04297-6

**Published:** 2025-06-05

**Authors:** Heba Abdelsalam, Harby Mostafa, Mohamed El-Ansary, Montaser Awad, Wael Sultan

**Affiliations:** 1 Agricultural and Biosystems Engineering Department, Faculty of Agriculture, Benha University, Moshtohor, Qalyobia Egypt; 2 Agricultural Research Center, Agricultural Engineering Institute, Giza, Egypt

**Keywords:** Magnetization, Salinity, Water properties, Agroecology, Plant stress responses

## Abstract

Salinity in irrigation water and soil poses a major challenge to the expansion of agricultural land in Egypt. High salt concentrations can lead to significant issues for both soil health and plant growth. Additionally, understanding water hydraulics in pressurized irrigation systems is crucial for their effective design, management, and operation. Magnetic water treatment has emerged as a potential solution to mitigate these salinity-related problems. This study was conducted to examine the effect of magnetic fields on the properties of irrigation water. Magnetic devices with two field intensities (1600 and 14,500 Gauss) were used to treat water at three salinity levels: tap water (219 ppm), 1000, and 2000 ppm. Magnetization was found to influence several physical and chemical properties of water, including velocity, dynamic viscosity, dissolved oxygen, surface tension, and pH. It also had a beneficial effect in reducing the total number of microorganisms. In contrast, electrical conductivity was not affected by magnetization. Variations in water velocity were influenced by both the strength of the magnetic field and the time elapsed after magnetization. Under magnetic treatment, water viscosity decreased, and surface tension dropped by 1.5 and 3% as salinity increased from 219 to 1000 and 2000 ppm, respectively. Additionally, the total number of microorganisms was reduced by 17.1 and 57.3% at 219 ppm, by 38.6 and 57.5% at 1000 ppm, and by 32.5 and 55.5% at 2000 ppm, under magnetic field intensities of 1600 and 14,500 G, respectively, when compared to non-magnetized water.

## Introduction

Due to climate change, more than 40% of the world’s population is currently experiencing water scarcity. To address this challenge, the use of low-quality water sources has become a practical solution in traditional irrigation systems^1^. In response to limited freshwater availability, reclaimed water containing moderate to high levels of pollutants, silt, and salinity has increasingly been used as an alternative in various irrigation practices^2,3^.

Magnetic treatment of saline water is an eco-friendly method of water treatment and crop irrigation. Magnetised water exhibits distinct mechanical, electromagnetic, and thermodynamic properties compared to conventional tap water. Because of its unique qualities, magnetised water is increasingly being used in a variety of applications, including industrial, environmental, medicinal, and agricultural domains, as magnetic devices become more advanced^4^. This treatment works by applying a magnetic field to alter the molecular structure of water, primarily by reducing hydrogen bonding between water molecules^5^. Magnetic water is also reported to exhibit a memory effect, with its altered properties lasting for approximately three days^6,7^. The fluctuation in water velocity influences the magnetic field of water and the time of magnetization. The variation in water velocity of 0.13 m/s resulted in a 4000G increase and a 2-hour extension of magnetization time^8^. Additionally, magnetic treatment has been found to increase the density and electrical conductivity of seawater while reducing its viscosity compared to untreated seawater^9^.

The application of a stationary magnetic field alters the physicochemical properties of water at intensities of 1000, 1500, and 2000 G^10^. The average contact angle of magnetized water was observed to increase to 0.262, 0.261, and 0.295 at these respective intensities, compared to 0.249 for tap water. The magnetic effect increased viscosity and reduced surface tension. Under the magnetic impact, the activation energy increased while the intramolecular energy of water decreased. As a result of the magnetic action, hydrogen bonds were formed, and the average size of the clusters increased. The EC, pH, and TDS of water all increased with the strength of the magnetic field^11,12^. As water flows through a magnetic field of increasing intensity, its properties fluctuate proportionally with the magnetic strength.

High salinity environments inhibit bacterial growth and reproduction, resulting in lower bacterial populations and consequently higher dissolved oxygen (DO) levels^13^. Magnetic treatment further enhances both pH and DO concentrations in water^14^. According to the principle of the reversed motor, the magnetic field increases electron density and DO concentration^15^. Experimental results showed that when water interacts perpendicularly with a magnetic field, the mechanical motion generates electrons, leading to changes in pH and DO levels in distilled water from 5.14 to 5.54 for pH and from 6.68 to 6.90 mg L^−1^ for DO. In addition to these effects, magnetization also alters the physicochemical properties of water by increasing its viscosity and decreasing surface tension^16^. The reduction in surface tension is strongly influenced by the strength of the magnetic field^17^. Surface tension reaches its lowest value when the magnetic field intensity ranges between 2000 and 3000 G, indicating the peak effect of magnetization. Beyond this range, the surface tension begins to increase again as magnetic intensity continues to rise.

Prior research has demonstrated that saline wastewater irrigation can boost soil bacterial community abundance^18^, however other investigations have found no discernible change or a declining trend. Once the magnetization process begins, the water molecule system undergoes a transformation after being exposed to a magnetic field for several hours, changing the system’s overall conformational energy. However, electrical conductivity is unaffected by magnetization. In addition, magnetization stability tests conducted in labs indicate that it lasts for 48 h^19^.

Water is capable of receiving signals from magnetic fields that directly influence biological cells and their essential functions^20^. In order to reduce microbiological water pollution, which clogs emitters in drip irrigation systems and reduces their uniformity, research is being done on the use of magnetic fields. Recent studies have demonstrated that magnetic field therapy may offer an effective, chemical-free solution for controlling biological clogging in drip irrigation systems^21^.

The objective of the present study was to assess how magnetism affects the hydraulic and design features of pressurized irrigation systems as well as their efficiency by altering the properties of low-quality irrigation water.

## Materials and methods

The present investigation was carried out at the National Irrigation Laboratory of Agricultural Engineering Research Institute (AEnRI), Dokki, Giza, Egypt, to study the effect of salinity and magnetic treatments on water properties.

The magnetization device was installed at the inlet of the magnetic water treatment subunit. The magnetic device is a product of Delta Water Co. for water treatment. The device is constructed from stainless steel, with an inner diameter of 5 cm, a length of 85 cm, and a weight of 11 kg. It supports a water flow rate of up to 25 m³/h and is designed to operate at temperatures up to 100 °C and pressures up to 15 bar. The device is effective for treating medium salinity water with concentrations up to 8000 ppm. With a magnetic capacity of 14,500 Gauss (1.45 Tesla), and another magnetic capacity 1600 Gauss (0.016 Tesla) with inner diameter 0.5inch and 50 cm length, water passes through the magnetic field and becomes magnetized, which causes some physical changes in the composition and shape of water molecules. Two main treatments consist of non-magnetic and magnetic irrigation water, and three sub-treatments of three salt concentrations including 219 ppm (tap water as control), 1000 and 2000 ppm. The salinity levels were prepared by adding Rashidy salt (containing about 99% NaCl, % Na = 31.64% and % Cl = 67.45%) and calcium carbonate to tap water to reach the required salinity.

A PVC beaker of 20 L capacity fitted at the top of the unit served as the storage tank for the untreated water. Water from the bottom of this tank enters the magnetic device. The device comprised of a pipe section with its internal diameter 50 mm. For the magnetic treatment of water, it was passed through the magnetic treatment device providing the water a magnetic field and collected in another PVC tank after the device. The experiment was done two times, one for each magnetic intensity with three replicates.

To measure the density of treatments, the magnetic water was placed in a 250 ml volumetric flask. Then, its mass was measured using sensitive balance.

Kinematic viscosity is the ratio of absolute viscosity in N-s/m^2^ to the density of the liquid in kg/m^3^. Mathematically, kinematic viscosity v (nu),

$$\:v=\:\frac{\mu\:}{\rho\:}$$

Where: *v* is kinematic viscosity (m^2^s^− 1^) and µ is dynamic viscosity of liquid, (pa.s)

For surface tension, the equipment has been made in laboratory to measure the surface tension of water, by using the capillary tube type as described by^12^. Surface tension is responsible for the phenomenon of liquid rising in capillary tubes; this phenomenon has been used to determine the surface tension of a liquid according to the following equation:

$$\:\gamma\:=\frac{1}{2}h\rho\:gr$$

Where: γ is surface tension coefficient (cm/dyne); r is the radius of the capillary tube (cm); h is height of the liquid in the capillary tube (cm); d is the density of the liquid (g/cm3); g is the acceleration due to gravity on Earth (cm/s²).

Electrical conductivity (EC) meter (ORION 105 Model, USA, 0 to 199.99 dS m^− 1^, and 0.5% F S accuracy) and pH meter (JENCO 1671 Model, USA, with 0.1 accuracy) were used to measure the electrical conductivity and pH of water.

Dissolved O2 was monitored during the study period at the lab using a dissolved oxygen portable (HI9142 dissolved oxygen meter). Regardless of the instrument, with different intensity of magnetic treatments and different salinity were measured in regard to dissolved oxygen (DO) in a unit of (%), considering temperatures (30).

For microbiological analysis of treatments, MacConkey Agar method was used according to^22^. A round dish with a diameter of 82 mm and a height of 11 mm in which MacConkey agar is placed. It is a culture medium designed for the growth of Gram-negative bacteria and their staining by lactose fermentation. MacConkey agar contains bile salts to inhibit gram-positive bacteria, Crystal violet (it also inhibits gram-positive bacteria) Neutral red (which stains lactose-fermenting).

Water samples are distributed by dipping the needle into the water sample, then dividing the dish into squares with the needle loaded with the sample, then making zigzag lines. The dishes are placed at a temperature of 32 degrees for 48 h. The total viable bacterial count was enumerated on plate ager medium at 32oC for 48 h. Coliform bacteria were enumerated on MacConkey ager medium for enumerated of coliform at 37^o^C for 24 h according to^22^. Colony-forming unit (CFU) is used in microbiology to estimate the number of viable bacteria or fungi in a sample.

A colony is a cluster of bacteria growing together. To measure the CFU, bacterial cultures are added to agar plates, often by serially diluting the original sample as it might be too concentrated to count. The number of visible colonies (CFU) present on an agar plate can be multiplied by the dilution factor to provide the CFU/ml value.

## Results and discussions

### Velocity

A reduction in water speed by increasing salinity was happened, from 1.43 m/s at 219ppm to 1.42 m/s at 1000 and 2000ppm (Table 1). While magnetization increased the speed from 1.43 to 1.45 and 1.46 m/s for 1600 and 14,500 G respectively, at 219 ppm. However, it increased with the same trend from 1.42 to 1.44 m/s for 1600 G and 1.45 m/s for 14,500 G at both 1000ppm and 2000ppm. The variation in the water velocity affects by the magnetic field of water and the time of magnetization as reported by^8^. The variation of water velocity by 0.13 m/s caused by an increase of 4000 G and extended the time of magnetization by 2 h^4^.

**Table 1:** Effect of magnetization process on water velocity (m/s).

Magnetic	Salinity (ppm)		
	219	1000	2000
Non magnetic	1.43	1.42	1.42
1600G	1.45	1.44	1.44
14500G	1.46	1.45	1.45

### Viscosity

The viscosity increased from 0.978 to 0.979 and 0.981with increasing salinity from 219 to 1000 and 2000 ppm as illustrates in (Table 2). By magnetically treating the water, a decrease in viscosity was observed. At an intensity of 1600 G, the viscosity decreased by 5.1% at 219 ppm, while at 1000 and 2000 ppm, the viscosity decreased by 4.3 and 1.7%, respectively. This means that magnetization reduces the viscosity. When the magnetic field strength was increased to 14,500 G, the viscosity decreased by 10.2, 6.8 and 3.3% at 219, 1000 and 2000 ppm, respectively, compared to the non-magnetized state. The higher the salt content, the higher the viscosity, and when the water was magnetically treated, the viscosity decreased. This result is consistent with the results of^9,10^, which show that under the influence of magnetism, the activation energy increases and the intramolecular energy of water decreases. As a result, hydrogen bonds are formed under the influence of the magnetic field and the average size of the clusters increases.

**Table 2:** Effect of magnetic and salinity on viscosity.

Magnetic	Salinity (ppm)		
	219	1000	2000
Non magnetic	0.978	0.979	0.981
1600G	0.953	0.952	0.950
14500G	0.902	0.918	0.934

### Density

As the salt content of water increases, its density also increases. When the salinity is 219, 1000 and 2000 ppm, the density is 0.97, 0.98 and 0.99 g/cm³, respectively, as shown in (Table 3). However, when water is exposed to a magnet, the density decreases, and as the magnetic field strength increases, the density also decreases. At 1600 G, the density is 0.96, 0.97 and 0.97 g/cm³, and at 14,500 G, the density is 0.94, 0.95 and 0.96 g/cm³.These results are similar with^9,23^.

**Table 3:** Effect of magnetization on water density (g.cm^− 3^).

Magnetic	Salinity (ppm)		
	219	1000	2000
Non magnetic	0.97	0.98	0.99
1600G	0.96	0.97	0.97
14500G	0.94	0.95	0.96

### Surface tension

**Fig. 1:**
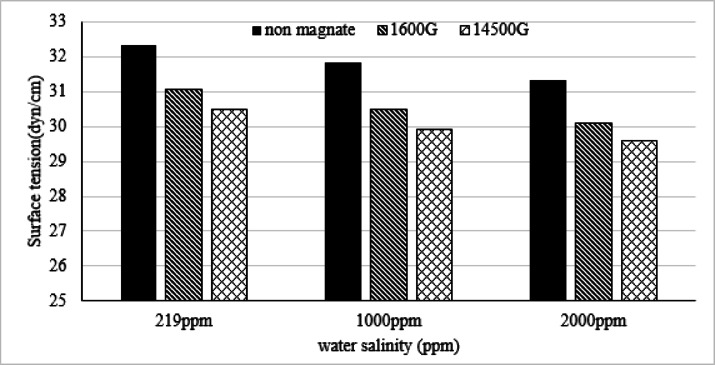
Water salinity and surface tension relationship under the influence of magnetism.

Results showed a decrease in the surface tension of water under the influence of magnetization and salinity (Fig. 1). When the water is not magnetized, the surface tension decreases by 1.5% when the salinity increases from 219 to 1000 ppm and by 3% when the salinity increases to 2000 ppm. When the water is exposed to a magnetic field of 1600 G, the surface tension of the water decreases by 4, 4.2, and 4% at 219, 1000, and 2000 ppm, respectively. When the magnetic field strength increases to 14,500 G, the attenuation rates are approximately 5.7, 6, and 5.6% at the same salinity, respectively. Al-Douri^10^ reported that magnetic field changes the molecules structure of water. The stationary magnetic effect changes the physicochemical properties of water at 1000, 1500, and 2000 G. The average contact angles are 0.262, 0.261, and 0.295 at strengths of 1000, 1500, and 2000 G, respectively, while the average contact angle of tap water is 0.249. The magnetic effect promotes viscosity and brought down surface tension. It was proved under magnetic effect; the activation energy was increased and energy of water intra molecular was decreased. As a result, under magnetic effect the bonds of hydrogen were created and mean size of clusters became larger.

### Electrical conductivity (EC)

The results showed that the EC value did not change when no magnetization treatment was performed, as shown in (Table 4). However, when the water was exposed to a magnetic field of 1600 G, the EC value increased by 17.4%, 26.9 and 30.6% after direct magnetization (time zero), 24 h and 48 h, respectively, which is equivalent to a salinity of 219 ppm. When the magnetic field strength was increased to 14,500 G, the EC value increased by 24.2% after magnetization (time zero). However, after 24 and 48 h, the EC value increased by 37.4 and 37%, respectively, which is equivalent to a salinity of 219 ppm. This result agreed with that obtained by^19,15^ who explained that the electrical conductivity increased due to the water structure being changed by the magnetic treatment. It is noticeable that with the increase in salinity, electrical conductivity also increased, and similarly, with the increase in magnetic flux density, electrical conductivity increased as well. However, over time during magnetization, the increase was slight as also mentioned by^24^.

**Table 4:** Influence of intensity and time after magnetization on the electrical conductivity at different salinities.

EC	219 PPM			1000 PPM			2000 PPM		
	0 h	24 h	48 h	0 h	24 h	48 h	0 h	24 h	48 h
Non magnet	219	219	219	1105	1105	1105	1901	1901	1901
1600 G	257	278	286	1109	1122	1117	1910	1929	1951
14,500 G	272	301	300	1110	1124	1124	1939	1943	1960

### Potential of hydrogen (pH)

**Fig. 2:**
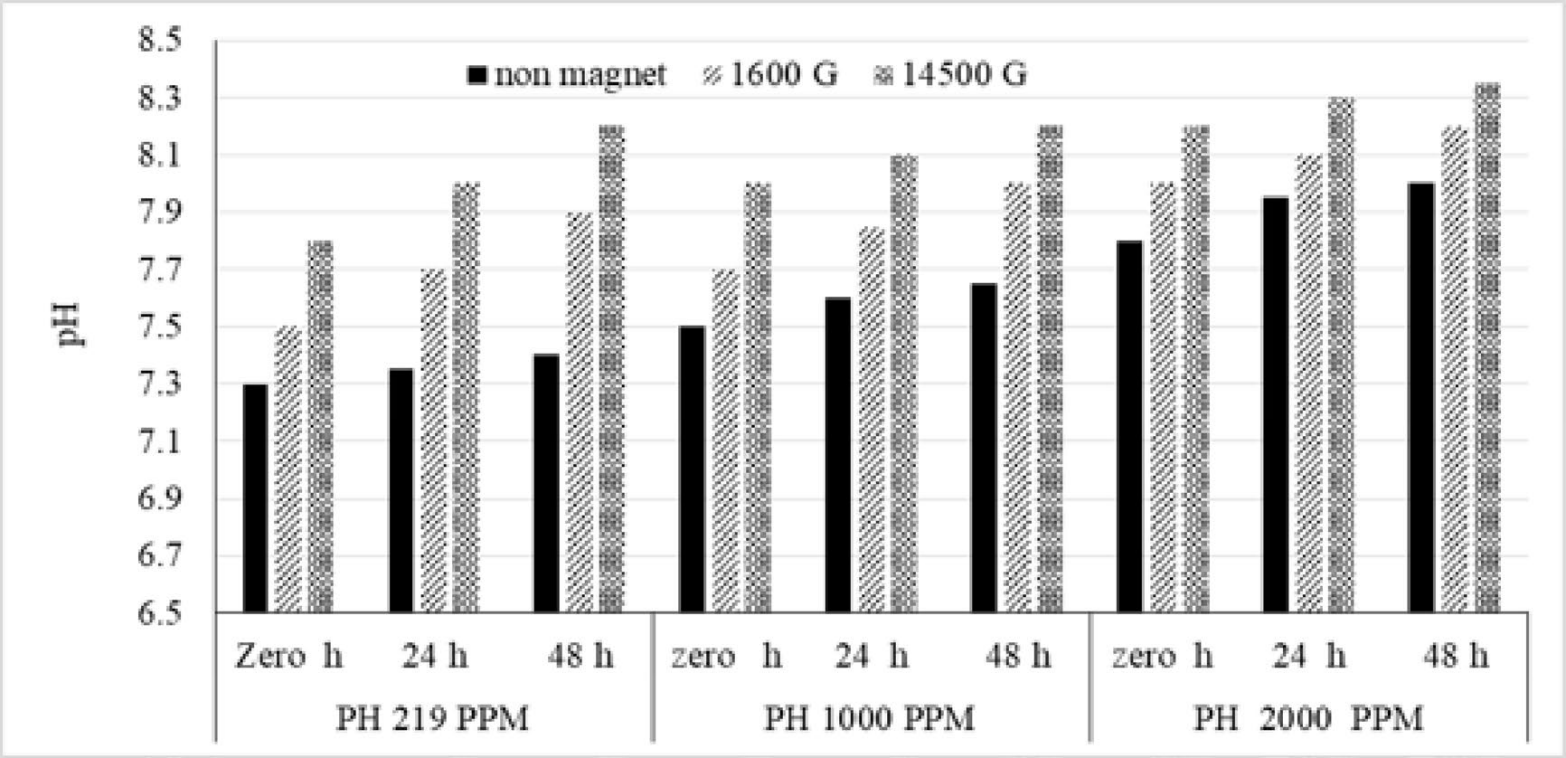
Influence of magnetization intensity, magnetization time, and salinity on pH.

As shown in Fig. 2, at 1600 G magnetization, the pH values ​​at 219, 1000, and 2000 ppm increased by 2.5, 2.7, and 2.6%, respectively, compared to the un-magnetized condition. When the magnetization strength was increased to 14,500 G, the pH values ​​increased by 6.8%, 6.6%, and 5.1%, respectively, compared to the un-magnetized water at the same salinity. Four magnets were used by^12^ with different size and intensity and noticed that the pH increased slightly over time, then return to its original value, confirming the memory of water effect.

Over time, when magnets were exposed to 1600 G strength after 24 h at salinities of 219, 1000, and 2000 ppm, magnetization increased pH by 4.7, 3.3, and 1.9%. After 48 h, pH rose by 6.8, 4.5, and 2.8% at the same salinity. Increasing the magnetic strength to 14,500 G also resulted in an increase in pH of 8.8, 6.6, and 4.4% after 24 h and of 10.8, 7.2, and 4.8% after 48 h, respectively. This is consistent with the observation that magnetic fields affect pH^11^. Once the process of magnetization starts, the molecular system of water is changed after several hours of exposure to magnetic field, which resulted in the alteration of the total conformation energy of the system, the observed changes in proton concentration value increases with the increment of intensity, reaching 17.22% as the water was exposed to intensity of 8000 G.

### Dissolved oxygen (DO)

As the salinity increases from 219 to 1000 and 2000 ppm, the percentage of dissolved oxygen increase from 79 to 82.4 and 84.1% respectively (Table 5). This results explained by^13^ who said with high salinity there is a lower quantity of bacteria as a result of salt content suppressing bacterial growth and multiplication, and thus high DO is produced, resulting in low biological oxygen demand. Prior research has demonstrated that saline wastewater irrigation can boost soil bacterial community abundance^18^.

However, when treating water magnetically at 1600 G, an increase in the DO percentage from 79 to 81.6, and it rises to 86.4 at 14,500 G was observed, with 0 time after magnetization. As time passes, the DO decreases where it drops from 81.6 to 76.5 and 63.1% after 24 and 48 h respectively, at 1600 G. This result agreed with^14^ who said that magnetization process increased pH and DO concentration while decreased the Oxidation and reduction potential (ORP) of water. Also^25^, explained that the DO increased due to the angle between the two hydrogen and oxygen atoms within the water molecule being lowered from 104° to 103° by the influence of the external magnetic field.

**Table 5:** Effect of magnetization intensities and time on DO with different salinities.

DO	219 ppm			1000 ppm			2000 ppm		
	0 h	24 h	48 h	0 h	24 h	48 h	0 h	24 h	48 h
Non magnet	79	75.2	74.6	82.4	76.2	67.5	84.1	70.7	65.1
1600 G	81.6	76.5	63.1	84	77.2	61.8	88.4	82.5	78.5
14,500 G	86.4	80.5	73.7	88.3	83.2	79.2	96.9	94.2	88.3

### Microbiological analysis

Magnetization was found to reduce microbial activity compared to conditions without magnetization (Table 6). At a salinity of 219 ppm, measured immediately after magnetization (zero time), the total microbial count decreased by 17.1% and 57.3% under magnetic intensities of 1600 G and 14,500 G, respectively. When salinity increased to 1000 ppm, microbial counts dropped by 38.6% and 57.5% at the same respective intensities. At a higher salinity of 2000 ppm, the reductions were 32.5% for 1600 G and 55.5% for 14,500 G.

The application of magnetic water or direct exposure of fungal colonies to a magnetic field showed positive results in inhibiting the growth of the selected fungi in the experiment^26,27^. Additionally, the effect of time on magnetized water was evident: at zero time (immediately after magnetization), bacterial counts were at their lowest compared to non-magnetized conditions. However, the number of bacteria increased after 24 h and doubled after 48 h.

## Conclusions

Magnetic technology can become a useful tool, as proved by many researchers, to tackle problems related to reduced crop productivity due to use of saline water in agriculture. Shrinking surface fresh water resources, increasing salinity of ground water, rising demand for water by urban and farming communities have made it difficult for planners to manage this precious resource to the satisfaction of all stakeholders. Therefore, this investigation was conducted to descript the effect of magnetic field on the properties of irrigation water. Magnetic devices with two intensities (1600 and 14500 G) were used to treat three saline water levels (tap water with 219 ppm, 1000 ppm and 2000 ppm). The effect of magnetization on velocity, viscosity, density, surface tension, electrical conductivity, water pH, dissolved oxygen content, and microbiological analysis were studied.

Magnetization was found to influence several water properties, including velocity, dynamic viscosity, dissolved oxygen, surface tension, and pH. It also had a positive effect in reducing the total number of microorganisms. However, electrical conductivity remained unaffected by magnetization. Both the strength of the magnetic field and the time elapsed after magnetization contributed to variations in water velocity. Under magnetic treatment, water exhibited reduced viscosity, surface tension, and microbial count. Therefore, magnetic treatment is recommended for saline water used in irrigation.

**Table 6:** Total count of microbes with varying exposure time to magnetism and different magnetism intensities.

Degree of salinity	Magnet intensity	Time (h)	Total number of microbes (cfu/ml)
219 ppm	Non magnet	Zero	11,700
		24	30,600
		48	57,300
	1600 G	Zero	9700
		24	27,800
		48	55,400
	14,500 G	Zero	5000
		24	16,900
		48	34,500
1000 ppm	Non magnet	Zero	25,900
		24	48,200
		48	78,900
	1600 G	Zero	15,900
		24	43,300
		48	75,300
	14,500 G	Zero	11,000
		24	35,200
		48	44,320
2000ppm	Non magnet	Zero	30,800
		24	52,100
		48	93,500
	1600G	Zero	20,800
		24	47,630
		48	89,500
	14,500 G	Zero	13,700
		24	40,700
		48	45,930

## Data Availability

All data generated or analysed during this study are included in this published article.
